# Towards an Epigenetic Treatment of Leiomyomas?

**DOI:** 10.1210/endocr/bqaa172

**Published:** 2020-11-06

**Authors:** Daniel Vaiman

**Affiliations:** Department of Development, Reproduction and Cancer, Cochin Institute, INSERM, CNRS, Université de Paris, Paris, France

**Keywords:** leiomyoma, epigenetics, animal model

Leiomyoma are very frequent benign tumors, affecting primarily smooth muscles, and especially the uterine muscle. They were described for the first time in 1793 by the clinician Matthew Baille (1761–1823). The frequency of leiomyoma makes them a major indication of hysterectomy. A 1990 study led to an estimated frequency of 77% of hysterectomies being motivated by leiomyomas (1), but the estimation varies hugely, from 5.4% to 77%, depending on the diagnostic approach or the population under scrutiny (2).

The initial culprits for such a disorder are suspected to be constituted of specific populations of stem cells, meaning that only a part of the cells found in the lesion are the source of its proliferation. Hence, populations of lowly differentiated cells that nevertheless retain a “memory” of their tissue of origin should be found in the lesions. Molecularly speaking, cell memory is based upon epigenetic mechanisms that enable a continuity when a given organ/tissue has to be regenerated or repaired from a pool of “stem cells.” Logically, epigenetic marks ought to be found in the subpopulation of stem cells present in a given organ or lesion; the most frequently studied epigenetic marks are deoxyribonucleic acid (DNA) methylation of cytosines present in Cytosine-Guanine (CG) dinucleotides.

 In the paper published in this issue of *Endocrinology* by Shimeng Liu and coworkers, the Bulun’s team explore a possible way of reducing leiomyoma lesions through a treatment by an epigenetic modulator, 5’-Aza-Cytidine (3). Building on their previous results and know-how (4, 5), the Bulun’s team was able to separate, from human samples, 3 cell populations by fluorescence assisted cell sorting (FACs) using CD34 and CD49: stem-cell like cells (LSCs), intermediate cells (LICs), and differentiated cells (LDs) that were globally evaluated by transcriptome and methylome analyses. Ribonucleic acid sequencing (RNA-seq) was used for the transcription analysis and MethylCap-Seq for the methylation analysis. This last technique is based upon the immunoprecipitation of a tagged Methyl-DNA binding protein with its bound DNA, followed by next generation sequencing. The paper finishes with an interesting in vivo study based upon a mouse model.

In terms of gene expression, the 3 types of cells present with the deregulation of specific gene sets. In particular, LDCs showed activation of transforming growth factor beta (TGFβ) pathways, indicative of differentiation and senescence, while LSCs harbor markers of signaling, cell cycle, and, unexpectedly, clusters of genes involved in the immune response. Interestingly, TET1, (as well as TET3 to a lesser extent) are major actors of genome demethylation and are expressed at a low level in LSCs, a bit counterintuitively, with the notion that demethylation is a feature of less differentiated cells, at least in embryonic development. However, when the genes of LSCs are forced into the “dogma” characteristics of 2 categories, “hypermethylation + down-regulation” and “hypomethylation + upregulation,” the latter category encompasses important clusters related to stem cell physiology. Indeed, the comparison between LSCs and the other 2 cell categories revealed gene-ontology terms strongly linked to stem cells, such as “stem cell endothelial differentiation during development,” “stem cell Notch signaling,” and “Stem cell response to hypoxia.” This observation suggests that this specific group of genes (demethylated and overexpressed) represent a driving force for maintaining an LSC population in the leiomyoma lesion, thus allowing its further developement. Locus-specific genomic analyses revealed differential methylation for loci important in cell differentiation/proliferation, such as at ESR1, TIMP3, ROR2, and MYH11, in association with their altered messenger RNA level, and a clear impact by 5’-Aza-Cytidine treatment (inducing genome demethylation). The in vivo part of the paper applied the 5’Aza Cytidine treatment to a mouse model of leiomyomas in comparison with a RU-486 treatment, a progesterone antagonist having previously been shown to be efficient to slowing down the growth of leiomyomas (6) that depends upon progesterone.

The mouse model is based upon a xenograft of human leiomyoma explants under the renal capsule that were previously treated or not with Ru486 or 5’Aza Cytidine (during 6 days). The evolution of the tumor was monitored 4 weeks after the graft; 5’-Aza induced a massive regression of the tumor size (36% of the size observed in the controls), while the decrease by Ru-486 treatment was much less spectacular (76% of the size observed in the controls). The authors pushed forward their analysis by analyzing through RNA-seq the effects of 5’-Aza on LSCs from samples collected from 3 patients. Interestingly, Aza treatment antagonizes mitotic division and gene silencing, while promoting epidermal cell differentiation and endocrine processes. In sum, this demethylating treatment tilts the gene balance towards cell differentiation, equating and mimicking the demethylation observed with this process. In the context of leiomyoma, this is consistent with the authors findings that leiomyoma stem cells are associated to TET1 low transcripts levels, and thus to rather high methylation. The 5’AZA treatment leading to demethylation is thus consistent with a shift from the LSC population towards the LSD population, slowing down the tumor growth by depletion of the stem cells. These results may suggest a track towards a treatment of the human disease.

## Data Availability

Data sharing is not applicable to this article as no datasets were generated or analyzed during the current study.

## Additional Information

***Disclosure Summary:*** The author has nothing to disclose.
